# Assessment of Copper and Zinc Levels in Hair and Urine of Children With Attention Deficit Hyperactivity Disorder: A Case-Control Study in Eastern India

**DOI:** 10.7759/cureus.20692

**Published:** 2021-12-25

**Authors:** Saurav Nayak, Suchanda Sahu, Suravi Patra, Joseph John

**Affiliations:** 1 Biochemistry, All India Institute of Medical Sciences, Bhubaneswar, IND; 2 Psychiatry, All India Institute of Medical Sciences, Bhubaneswar, IND; 3 Pediatrics and Child Health, All India Institute of Medical Sciences, Bhubaneswar, IND

**Keywords:** copper/zinc ratio, zinc, copper, adhd, icpoes, heavy metals

## Abstract

Background

Attention deficit hyperactivity disorder (ADHD) is a neuro-developmental ailment diagnosed with inattention and hyperactivity-impulsivity. It is one of the most prevalent neurodevelopmental disorders and has complex aetiology, both genetic and environmental. There is a perceived decrease in skill acquirement, leading to insufficient income and job opportunities as adults, which drives them towards poor physical and mental outcomes compared to their contemporaries without ADHD. The impact of heavy metals on ADHD is a topic of interest but is much less studied. Copper has been implicated as a pro-oxidant and in the metal accelerated production of free radicals that may affect oxidative stress. Zinc also serves as an antioxidant, and changes in its concentrations may impact the homeostasis of oxidative stress.

Methods

Twenty-four children diagnosed with ADHD were taken as cases and matched with 24 healthy controls. Hair and urine samples were collected from all the study participants. The samples were collected in sterile containers according to established protocols. Acid digestion of hair samples was done using 65% nitric acid and 30% hydrogen peroxide. Urine samples were extracted by a solution of 0.1% Triton-X-100 and 1% ultrapure nitric acid. The levels of zinc and copper were determined in both samples by inductively coupled plasma optical emission spectrometry (ICP-OES). The copper/zinc ratio (Cu/Zn) was calculated from these values. Mann Whitney U Test and receiver operating characteristic (ROC) analysis were done to estimate statistical significance.

Results

The median age of the study population was eight years. Overall, 34 male and 14 female subjects participated. There was no significant difference in height, weight and BMI between the cases and controls. Hair zinc levels in the ADHD group (198.49 µg g^-1^ of hair) was significantly lower than the control group (527.05 µg g^-1^ of hair). However, hair copper levels were increased significantly in the ADHD children (14.01 µg g^-1^ of hair) compared to the controls (7.43 µg g^-1^ of hair). Urine zinc levels were significantly lower in cases than controls (525.7 µg g^-1^ of spot urine creatinine vs 1374.09 µg g^-1^ of spot urine creatinine). However, copper levels in urine were higher in the ADHD children (17.01 µg g^-1^ of spot urine creatinine compared to 7.26 µg g^-1^ of spot urine creatinine in controls). Both hair and urine copper to zinc ratio was significantly higher in the ADHD group. On ROC analysis, the hair Cu/Zn ratio had an area under the curve (AUC) of 0.920 (p-value <0.001), and the urine Cu/Zn ratio had an AUC of 0.967 (p-value <0.001). When used as a diagnostic classifier for ADHD based on the cut-off value determined by ROC, both hair and urine Cu/Zn ratio had high sensitivity and specificity.

Conclusion

Low zinc levels in the urine and hair of children and higher levels of copper may impact the aetiology of ADHD in these children. At an early stage, the Cu/Zn ratio in both hair and urine samples may be used as a precise biomarker to identify and monitor such children.

## Introduction

According to the World Health Organization (WHO), neurodevelopmental diseases are today's most significant public health challenges accounting for a significant component of this category, impacting morbidity, mortality, disability, and quality of life [1]. According to the Diagnostic and Statistical Manual of Mental Disorders (DSM-5), attention deficit hyperactivity disorder (ADHD) is a neuro-developmental ailment diagnosed with inattention and hyperactivity-impulsivity, which impedes the natural functioning and growth of the child [2]. The prevalence rate of ADHD varies widely, although Polanczyk et al. reported a rate of 9.5% in a meta-analysis to assess global prevalence [2]. As reported by Jaisoorya et al., there is a 1.5% prevalence of ADHD in most Indian communities [3], whereas another study by Juneja et al. ranks it to as high as 7.2% [4]. Ranging from 2:1 to 9:1, Rucklidge reports a higher prevalence in boys than girls [5]. The short span of attention (Inattentiveness) presents with cardinal signs of easy distractions, making careless mistakes, constantly changing activities, and a general inability to listen to or carry forward with instructions. Hyperactivity and impulsiveness present primarily in children with them being unable to stay calm and still with a constant fidgeting behaviour with excessive physical movement. Frequently scoring low in tests, being considered bad or slow in a study by peers, being expelled/suspended from classes are issues associated with ADHD children [6]. Apart from these, there is also a perceived decrease in skill acquirement, leading to insufficient income and job opportunities as adults, which drives them towards poor physical and mental outcomes compared to their contemporaries without ADHD [1]. It is well perceived that genetic factors affect 60-80% of the overall ADHD cases. Still, gene-environment interaction and specific environmental influences might mediate the aetiology substantially [7].

Several studies highlight the role of oxidative stress as an important participating agent in the aetiology of ADHD. An increase in the production of free radicals and oxidative stress may be affected by and affect the homeostasis of certain trace elements [8]. Trace elements such as copper and zinc play an important role in pathways concerning oxidant and antioxidant mechanisms. Therefore, altered levels of these elements and their imbalance may lead to increased susceptibility to oxidative damage of important cellular components, which may contribute to the pathogenesis of ADHD.

Copper is required for the catalytic activity of many enzymes involved in the neurophysiology of this disorder, including Cu/Zn superoxide dismutase (antioxidant protection of cells), tyrosinase and dopamine-hydroxylase (metabolism of dopamine, noradrenaline, and epinephrine), monoamine oxidase (catecholamine degradation), and ceruloplasmin (iron homeostasis in the brain). In several studies, copper has been implicated as a pro-oxidant and in the metal accelerated production of free radicals. As a result, high Cu levels and copper-mediated neurotoxicity may be linked to the creation of a copper-dopamine complex, which is followed by dopamine oxidation. This condition has been linked to physical and mental exhaustion, depression, and other mental illnesses like schizophrenia, learning impairments, ADHD, and general behavioural issues [9,10].

Zinc is a critical component in the metabolism of neurotransmitters, prostaglandins, and melatonin and has an indirect effect on dopamine metabolism. It is required to form a variety of metalloenzymes and metal-protein complexes, most notably in the central nervous system, and hence contributes to the brain's structure and function. Additionally, the dopamine transport system contains a zinc-binding site required for the brain's transport systems [11]. Zinc also serves as an antioxidant in the body, notably in the brain, by shielding the sulfhydryl groups of proteins and enzymes from free radical damage. This element may also affect the foetus's cell division, maturation, and growth, as well as the child's neurodevelopment and intellect later in life. As a result, changes in Zn metabolism in response to oxidative stress may have a role in developing neurological dysfunctions [12,13].

Hair trace elements may have a relationship with these elements' storage in the body. Hair analysis has been used to determine exposure to several environmentally hazardous substances based on this concept [14]. Urine analysis also presents a unique non-invasive method to assess heavy metals in circulation as it has a high degree of correlation with the corresponding serum levels. With these caveats in mind, hair and urine concentrations of the copper and zinc were estimated.

The aim of the study was to emphasize the levels of copper and zinc in hair and urine of ADHD children. We hypothesized that there is an existent difference in these levels when compared to healthy children and thus the study was conducted.

## Materials and methods

Study design

This was a case control study carried out at All India Institute of Medical Sciences, Bhubaneswar (Odisha, India), between August 2019 and February 2021. Ethical clearance for the study was duly permitted by the Institutional Ethical Committee of All India Institute of Medical Sciences Bhubaneswar vide letter no. IEC/AIIMS BBSR/PG Thesis/2019-20/01, dated August 05, 2019. The requisite Written Informed Consent and Written Informed Assent were taken from the subjects participating in the study following the Indian Council of Medical Research, National Ethical Guidelines for Biomedical Research involving Children. All participants and parents were provided with a written Participants' Information Sheet, that contained the details of the study hypothesis, design, sample collection methodology and a clear statement about the confidentiality and anonymity of their data. The children were aged between three and 16 years and were diagnosed by a medical psychiatrist following DSM-5 Criteria for ADHD. Age- and sex-matched controls were recruited. Any children with intellectual disability, any neurological or behavioural disorder, or suffering from any chronic illness were duly excluded. Sample size was calculated, on OpenEpi Sample Size Calculator for Case Control Study, by the method for rates and proportions as prescribed by Fleiss with continuity correction, from existing data, to be 48 [15]. 

Sample collection and processing

Convenience sampling was done to get the samples for the study. Those subjects who were enrolled as ADHD subjects in the hospital registers were contacted and samples were collected.

Hair samples were collected from the nape of the children by alcohol-washed stainless steel scissors, approximately amounting to 500mg [16]. These were collected in containers pre-treated with nitric acid and stored at -80^0^C for extraction. Hair samples were digested in a solution of 6mL of 65% nitric acid and 2mL of 30% hydrogen peroxide by heating at 180^0^C for 15 minutes, followed by cooling at room temperature for 15 minutes [17]. Urine samples were collected as first morning urine sample collected by mid-stream clean catch technique. About 50mL of urine was collected in a pre-treated container and stored at -80^0^C. The extraction of heavy metals from urine samples was done using a solution containing 0.1% (V/V) Triton-X-100 (Sigma, Burlington, MA, USA) and 1% ultrapure concentrated nitric acid [18].

The measurement of heavy metal levels was done by Avio™ 200 dual-view instrument equipped with Syngistix™ software for inductively coupled plasma optical emission spectrometry (ICP-OES) (PerkinElmer, Waltham, MA, USA). Both axial and radial views were used depending on the concentration of the heavy metal to be analysed. Calibration standards and blanks were used for analysis for samples for each heavy metal. Urine creatinine was measured from the collected sample by Jaffe's Method in a Beckman Coulter (Brea, CA, USA) AU480 autoanalyser. Hair heavy metal levels were expressed as microgram per gram of hair (µg g^-1^ of hair) and urine heavy metals as microgram per gram of spot urine creatinine (µg g^-1^ of spot urine creatinine).

Demographic and anthropometric data were also collected from the subjects. All data were coded and stored electronically. The data was completely anonymised and was used only by the authors for data analysis without any identifiers.

Statistical analysis

The dataset was subjected to the Shapiro-Wilk test for normality of data. Parametric data were expressed in the form of Mean ± SD and non-parametric data expressed in Median (IQR). Independent sample's t-test and Mann Whitney U test were used to determine the difference between the groups. Receiver operating characteristic (ROC) and area under the curve (AUC) analysis were done to check for diagnostic and predictive values of the heavy metals. All data analysis was done using Statistical Package for Social Sciences (SPSS) version 19.0 (IBM Corp., Armonk, NY, USA) and JASP v0.13. A p-value of <0.05 was considered statistically significant.

## Results

The data was found not to have a normal distribution, hence non-parametric methods were used to express the statistical analysis. The median age of the study population was eight years of age. Most of the subjects were male (72.92%) and had a median BMI of 20.46. There was no significant difference in the children's weight, height, and BMI in the two groups. The demographic data have been summarised in Table 1.

**Table 1:** Demographic profile and comparison of the study population a: Chi Square test b: Mann Whitney U test

Parameters		Overall Population (n = 48)	Cases (n = 24)	Controls (n = 24)	p-Value
Sex	Male, n (%)	35 (72.92%)	17 (70.83%)	17 (70.83%)	0.745 ^a^
	Female, n (%)	13 (27.08%)	7 (29.17%)	7 (29.17%)	
Age in years, Median (IQR)		8 (6.5 - 10)	8 (6.5 - 10)	8 (6.5 - 10)	0.739 ^b^
Height in cm, Median (IQR)		120 (110 - 135.5)	121.5 (112.9 - 137.5)	117.5 (110 - 135)	0.391 ^b^
Weight in kg, Median (IQR)		32.5 (24.5 - 38)	35 (25.5 - 39.5)	29 (23.5 - 38)	0.591 ^b^
BMI		20.46 (19.13 - 22.88)	19.76 (17.51 - 23.49)	20.84 (19.88 - 22.38)	0.332 ^b^

The hair zinc levels were lower in ADHD cases as compared to the controls. The median level in cases was 198.49 µg g^-1^ and 527.05 µg g^-1^. However, copper levels were significantly increased in the ADHD group compared to (14.01 µg g^-1^ of hair and 7.43 µg g^-1^ of hair, respectively). Urinary concentration of zinc was also found to be significantly lower in the ADHD group. Median urine zinc levels were 848.9 µg g^-1^ of spot urine creatinine lesser in the ADHD cases as compared to the healthy controls. However, urine copper levels were significantly higher in ADHD. The data has been summarised in Table 2.

**Table 2:** Comparison of heavy metal levels in hair and urine samples between the cases and controls

Sample	Heavy Metal	Cases, Median (IQR)	Controls, Median (IQR)	p-Value
Hair (in µg g^-1^ of hair)	Zinc	198.49 (124.95 - 248.12)	527.05 (404.19 - 1193.94)	<0.001
	Copper	14.01 (7.6 - 20.45)	7.43 (4.31 - 14.72)	0.013
Urine (in µg g^-1^ of spot urine creatinine)	Zinc	525.7 (199.52 - 962.2)	1374.09 (881.63 - 1808.84)	<0.001
	Copper	17.01 (8.53 - 40.79)	7.26 (4.27 - 12.56)	0.007

**Figure 1:**
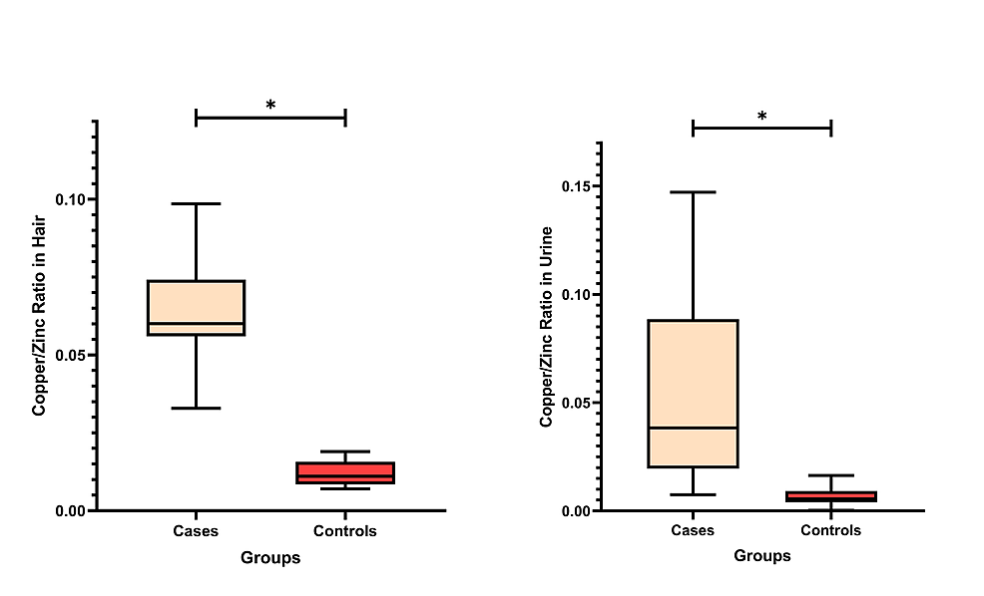
Difference in Copper/Zinc ratio between cases and controls in hair and urine samples * p-value <0.05

The copper to zinc ratio in hair significantly differed between the groups (p<0.001), as mentioned in Figure 1. The median hair copper to zinc ratio was in the ADHD cases 0.0601 (0.0561 - 0.0737) and 0.0110 (0.0085 - 0.0154) in the control group. In the case of urine samples, the median copper to zinc levels in the ADHD group was significantly higher (p<0.001). The median level in the ADHD group was 0.0384 (0.0198 - 0.0863) compared to that of the controls, i.e., 0.0056 (0.0041 - 0.0090).

**Figure 2:**
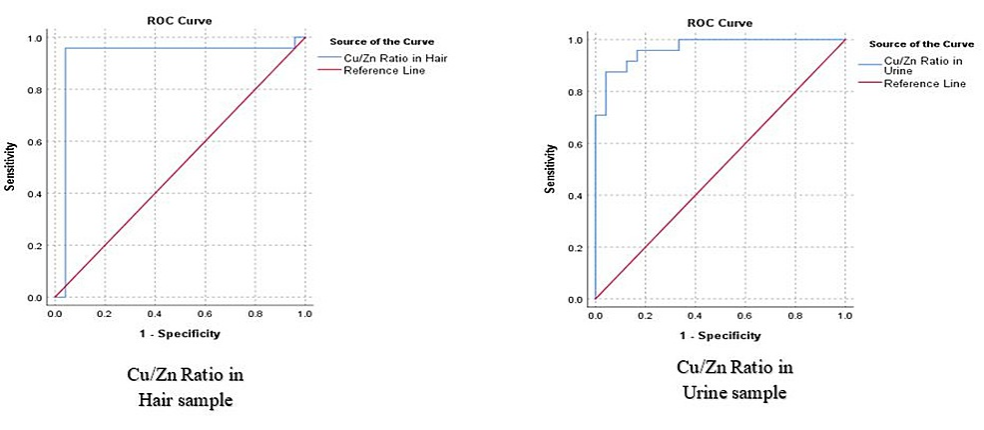
ROC Analysis for Cu/Zn Ratio in Hair and Urine Samples

The classification accuracy of heavy metals was determined by ROC analysis. Cu/Zn ratio in hair samples had a very high classification accuracy (AUC = 0.920, p-value <0.001) as compared to zinc (AUC = 0.151, p-value = 0.059) and copper (AUC = 0.710, p-value = 0.005). Similarly, in the urine sample also Cu/Zn Ratio had a very high classification accuracy (AUC = 0.967, p-value < 0.001). Urine zinc was insignificant in classification (AUC = 0.214, p-value = 0.069) whereas copper had a comparatively lower efficacy (AUC = 0.727, p-value = 0.002) as compared to Cu/Zn Ratio. The ROC curves have been depicted in Figure 2.

From the ROC analysis with the aid of the Youden Index, cut-off values were estimated for the Cu/Zn ratio for both hair and urine samples. For hair Cu/Zn ratio the cut-off was determined to be 0.0274 (Youden Index = 0.917, Sensitivity = 95.83%, Specificity = 91.67%). A value of 0.0164 was determined as the cut-off for urine Cu/Zn ratio (Youden Index = 0.833, Sensitivity = 87.5%, Specificity = 95.83%). The classification was used to determine the predictive probability of the Cu/Zn ratio for ADHD cases. Hair Cu/Zn Ratio had a Positive Predictive Value of 92% and Negative Predictive Value of 95.65%. Similarly, the urine Cu/Zn Ratio had a Positive Predictive Value of 95.45% and a Negative Predictive Value of 88.46%. 

## Discussion

Our study aimed at studying the levels of zinc and copper in children diagnosed with ADHD and determining if there was any significant difference compared to the healthy children who have not been diagnosed with ADHD. We found out that there exists a significant difference in the levels of zinc as well as copper in the hair and urine samples of the studied children.

In studies, copper deposition has been linked to localised brain oedema, neurodegeneration, nerve fibre demyelination, and glial cell proliferation. Necrosis, cystic transformation, interstitial focus, and cavernous vacuolation are all possible risk factors for the development of these illnesses and may contribute to the origin of ADHD in children [19]. Studies on children have reported increased serum copper levels, as evidenced by higher hair copper levels [20,21]. Our study, however, estimated an increase in the hair and urinary copper levels in the ADHD group. These findings corroborate recent research by Goodlad et al. and Huang et al. that found comparable elevated levels in the ADHD population [22,23].

Zinc is required for brain and neural tissue formation and growth since it transforms dietary pyridoxine to its active form (i.e., pyridoxal phosphate). Additionally, pyridoxine is required for the conversion of tryptophan to 5-hydroxytryptamine, which is associated with ADHD. If prior literature is to be believed, the evidence for zinc and ADHD is rather mixed. Skalny et al. and Tippairote et al. examined hair zinc levels and discovered that they were significantly higher in the ADHD group [21,24]. However, our study found a decisive level of evidence for lower hair zinc levels and very strong evidence of lower urinary zinc in the ADHD group. Our findings were in accordance with the studies done by Bilici et al. and Quist and Kennedy, who have reported significantly lower levels of hair and urine zinc levels in the ADHD population of the study [25,26].

From this study, it can be considered that Cu/Zn ratio is an important parameter in assessing a child with ADHD. A similar manifestation was determined by Viktorinova et al. in a population of Russian children, in which they had found significantly higher levels of Cu/Zn levels in serum [8]. A deranged copper-zinc ratio may be associated with a decreased capacity of the organism to maintain or regenerate copper-zinc homeostasis following the influence of destabilising events on these elements' metabolism.

It has been suggested that disruption of the dopaminergic, serotonergic, and noradrenergic neurotransmitter systems may contribute to the symptoms of a number of mental and neurological illnesses [27]. Copper and zinc levels must be optimal for the metabolism and activity of neurotransmitters. Copper is required for the action of several enzymes involved in the metabolism of neurotransmitters, including dopamine hydroxylase and monoamine oxidase. Copper can cause dopaminergic neurons to die by damaging their antioxidant defence system, as demonstrated in animal experiments [9].

Zinc deficiency has been linked to DNA damage, which may be caused by a combination of oxidative stress, impaired antioxidant defences, and defective DNA repair [28]. Zinc deficiency, when combined with copper toxicity, may result in dysregulated neurotransmitter system function, decreased Zinc finger protein activity, and decreased zinc-dependent gastrointestinal enzymatic activity. Zinc deficiency can result in oxidative stress, which can affect Zinc finger patterns important in cell signalling as transcription factors [29]. Additionally, zinc deficiency is associated with synaptic dysregulation, and the majority of candidate genes for autism have been discovered in excitatory post-synapses. Finally, zinc has been linked to GABA and glutamate control, and zinc deficiency has been linked to GABAergic dysfunction [30].

There were several limitations associated with our study. The study was focused on the eastern Indian population; however, due to the ongoing coronavirus disease 2019 (COVID-19) pandemic, the subjects recruited got strictly confined to only one state and clustered around the city of the tertiary care hospital. Multiple environmental factors are potent risks for the aetiology of ADHD in children, and studying each confounder is a cumbersome process. Hence, this study concentrated only on the two heavy metals. Detailed antenatal, postnatal, and other family and socioeconomic histories were not obtained due to the constraints of time and contact, which might affect the diagnosis of ADHD in the children.

## Conclusions

Results have suggested a lower zinc level in hair and urine of ADHD children as compared to the healthy control group. However, the median copper levels are higher in both samples of ADHD children. Cu/Zn ratio for hair and urine is significantly higher in the ADHD group. Cu/Zn ratio can be effectively used as an effective predictor of ADHD diagnosis in children and thus can potentially be used as a tool for assessing ADHD children and monitoring their progression of management. Also effective use of zinc supplementation and copper chelation can be further studied as treatment modalities for ADHD.
